# Protective Effects of Intranasally Administrated Oxytocin-Loaded Nanoparticles on Pentylenetetrazole-Kindling Epilepsy in Terms of Seizure Severity, Memory, Neurogenesis, and Neuronal Damage

**DOI:** 10.1021/acschemneuro.2c00124

**Published:** 2022-06-17

**Authors:** Hakan Sahin, Oguz Yucel, Serkan Emik, Gozde Erkanli Senturk

**Affiliations:** † Department of Histology and Embryology, Cerrahpasa Faculty of Medicine, Istanbul University—Cerrahpasa, Istanbul 34098, Turkey; ‡ Department of Chemical Engineering, Faculty of Engineering, Istanbul University—Cerrahpasa, Istanbul 34320, Turkey

**Keywords:** pentylenetetrazole, epilepsy, intranasal administration, oxytocin, nanoparticles, histology

## Abstract

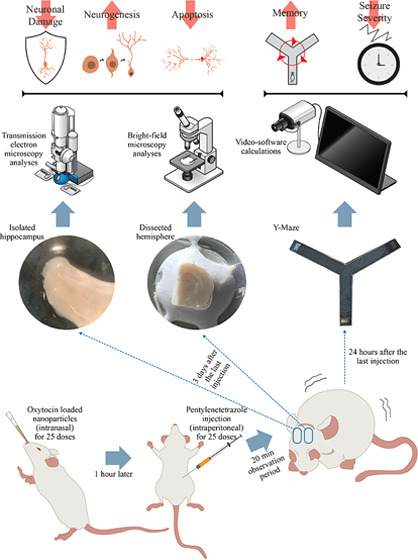

Pentylenetetrazole (PTZ)-induced kindling is an animal model for studying human temporal lobe epilepsy (TLE), which is characterized by alterations of hippocampal neurons and memory. Although the intranasal (IN) administration of oxytocin (OT) has limited efficiency, nanoparticles (NPs) are a promising candidate to deliver OT to the brain. However, there are very limited data on epilepsy research about oxytocin-loaded nanoparticles (NP-OTs). The aim of this study is to investigate the effects of IN administration of chronic NP-OTs on the hippocampus of PTZ-induced male epileptic rats in terms of seizure severity, memory, neurogenesis, and neuronal damage. Saline/OT/NP-OTs were administrated to both control (Ctrl) and PTZ groups intranasally. Consequently, saline and PTZ were injected, respectively, 25 times every 48 h. Then, seizure severity (score and latency) was calculated for the PTZ groups. A spatial working memory evaluation test (SWMET) was performed after the last injection. Hippocampus histopathology, neurogenesis, and apoptosis were demonstrated. Serum total antioxidant status (TAS) and total oxidant status (TOS) levels and the oxidative stress index (OSI) were measured. We showed that OTs and NP-OTs prevented the kindling development and had positive effects on seizure severity. SWMET-related behaviors were also recovered in the PTZ + NP-OT group. A significant increase of neurogenesis and decrease of apoptosis in the hippocampus of the PTZ + NP-OT group were observed, while OTs and NP-OTs had protective effects against PTZ-induced damage to hippocampal neurons. Our results indicate that the chronic administration of NP-OTs may have positive effects on hippocampal damage via increasing neurogenesis and decreasing apoptosis and seizure severity.

## Introduction

The hippocampus is a part of the limbic system in the brain that performs many tasks such as learning, memory, location, and emotional behavior. This structure consists of different subunits such as the cornu ammonis (CA) and the dentate gyrus (DG). Especially the DG region where the adult neurogenesis occurs has been associated with various pathophysiological events including cognitive dysfunctions.^1−3^ The hippocampus is involved in many different types of central nervous system (CNS) pathologies such as temporal lobe epilepsy (TLE). TLE is a type of focal epilepsy, which is characterized by complex seizures that show high resistance to antiepileptic drugs (AEDs). In addition to the seizures, patients may suffer from cognitive dysfunctions such as memory impairment.^4−6^ Those cognitive dysfunctions may be caused by adult neurogenesis alterations, which are seen in both human TLE and experimental animal models.^7,8^ Although AEDs are used to suppress seizures, there is no curative treatment approach for memory problems. On the contrary, it is known that some AEDs can worsen these cognitive functions as a side effect.^9^

Oxytocin (OT) is a neuropeptide hormone synthesized from the hypothalamus in mammals. This hormone is associated with many different physiological events both in the CNS and the peripheric organs. Researchers have shown that the exogenous administration of OT had beneficial effects on some CNS abnormalities.^10−14^ However, a major problem is the way of administration considering the blood–brain barrier (BBB).^15^ Thus, it is usually preferred to apply this neuropeptide by the intranasal (IN) route, which is a noninvasive method with a higher rate of passing through the BBB compared to the other systemic administrations.^16,17^ By IN OT administration, only a small portion of the peptide might pass to the CNS, despite the rate being still higher compared to the other systemic administrations (e.g., intraperitoneal and intravenous injections). It is considered that only the accumulation of OT in the subarachnoid space may have the ability to pass through the BBB by a nonspecific pathway due to the difference in the concentration. Another aspect of OT administration is the short half-life, which is almost 30 minutes in the cerebrospinal fluid and only a few minutes in the blood circulation when administered exogenously.^18^ Taking these into consideration, previous studies suggested that the development of new noninvasive methods is needed to make OT administration more effective. OTs have recently been applied to the brain with nanoparticle (NP)-carrying systems, and anticipative results were seen both in vitro and in vivo.^19,20^ Hereby, nanomedicine, which requires a multidisciplinary field of study, can lead us to success in this regard.^21,22^ Moreover, IN-administered NP-encapsulated OTs demonstrated more effective outcomes on epileptic mice compared to the mice treated with OT alone.^23^

Pentylenetetrazole (PTZ) kindling is a representative experimental rodent model for the human TLE as it exhibits acquired spontaneous seizures, damaged hippocampal neurons, altered neurogenesis, and impaired memory^7,24,25^ in the animals. Furthermore, it has been shown that only high-dose and short-term administration of OT may have some positive effects on acute seizure models, but its effects on PTZ-kindling have not yet been reported.

This study aims to determine the effects of IN-administered oxytocin-loaded nanoparticles (NP-OTs) on seizure severity, spatial memory, and hippocampus histology in PTZ-injected rats when compared to OT administration alone.

## Results and Discussion

### Chemical Structure, Particle Size, and Release Profile of Oxytocin-Loaded Nanoparticles

**Figure 1:**
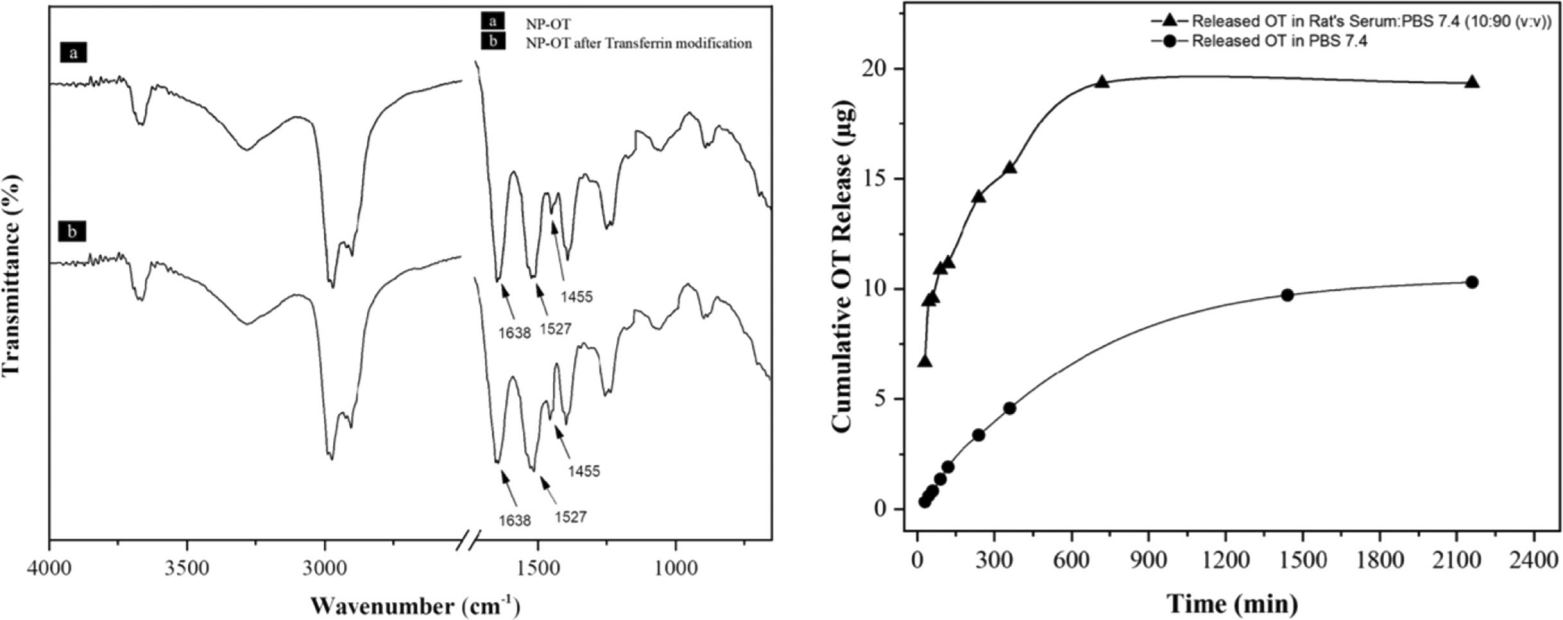
Chemical characterization of NP-OT and OT release profile from NP-OTs. FTIR spectra (left): (a) spectrum of NP-OTs and (b) spectrum of NP-OTs after transferrin modification. Release profile from phosphate-buffered saline (PBS) and PBS with 10% rat serum (PBS/rat serum 90:10 (v/v)).

The chemical structure characterization of synthesized NP-OTs was performed by Fourier transform infrared (FTIR) analysis, and the collected spectrum is shown in Figure 1a. The characteristic bovine serum albumin (BSA) peaks (cm^–1^) of NP-OTs are as follows: 1638, amide I (C=O stretching vibrations); 1527, amide II (N–H bending vibrations); and 1455, amide III (C–N bending vibrations). As seen in the figure, after the modification by transferrin (Figure 1b), a significant increase in the peak intensities of the amide I and amide II vibrations was observed, indicating a successful conjugation.^20^

**Figure 2:**
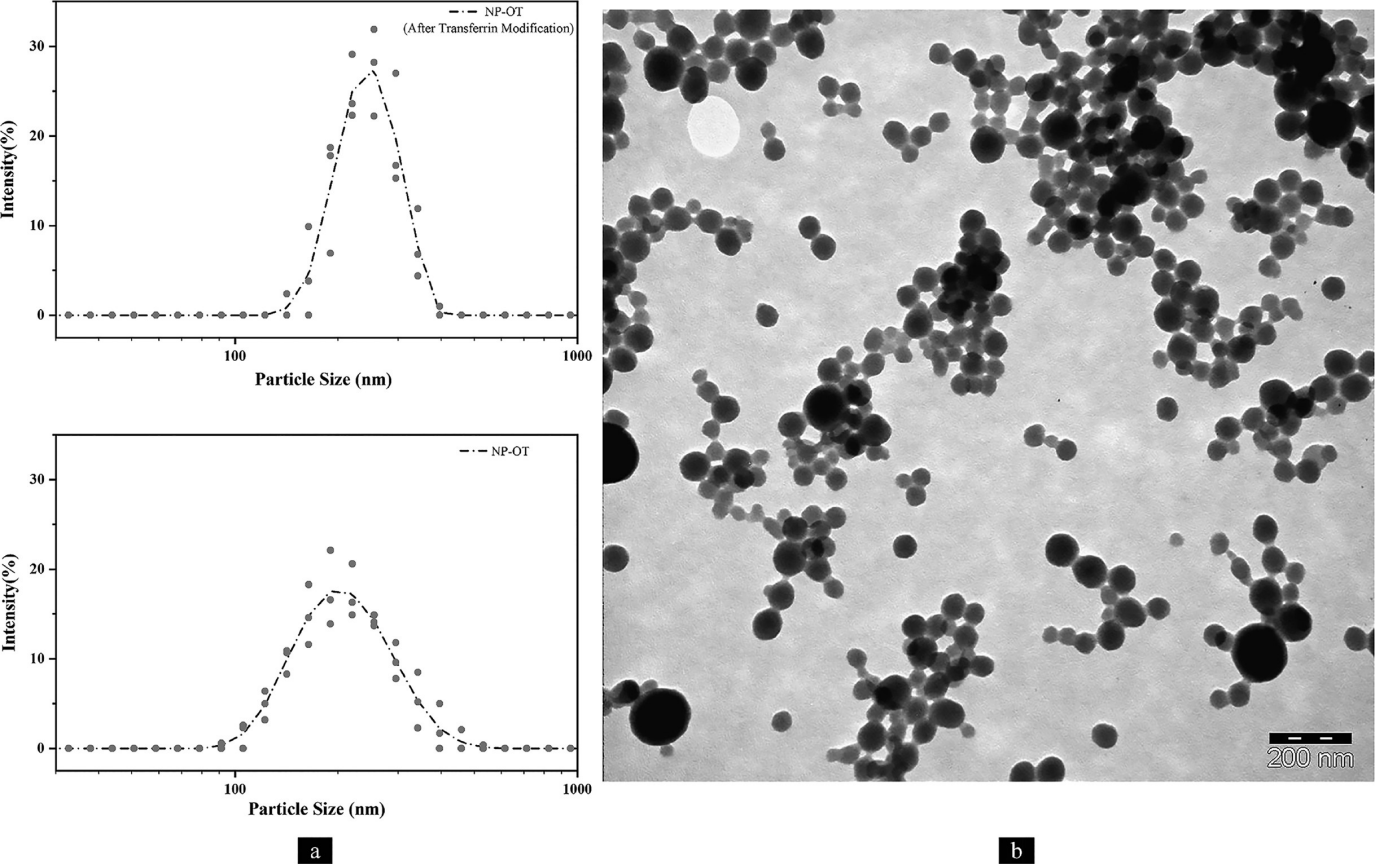
NP-OT size and TEM measurements. (a) Average particle size of NP-OTs (bottom) and the average particle size of NP-OTs after transferrin modification (top). DLS measurement was performed in an aqueous solution at 25 °C at a 90° scattering angle. (b) TEM measurement for NP-OTs (scale bar 200 nm).

Dynamic light scattering (DLS) measurements determined the particle size of oxytocin-loaded nanoparticles before and after transferrin modification, and the obtained results are illustrated in Figure 2a. The average particle sizes of oxytocin-loaded nanoparticles and NP-OTs (after transferrin modification) were determined to be 214 ± 76 and 236 ± 34 nm, respectively. As an expected result, due to the attachment of a new molecule to the main structure, the particle size of the NP-OTs slightly increased after transferrin modification. Transmission electron microscopy (TEM) results illustrated in Figure 2b also confirmed the size of the nanoparticles found by DLS measurements.

The calculated cumulative drug release amounts from in vitro release studies performed in two different release mediums (PBS and 10% rat serum with PBS) are given in Figure 1. As seen in the figure, the drug release in the 10% rat serum medium was significantly higher and faster than that in PBS, indicating an effective drug release, and within the 1st hour, they were found to be 0.84 and 9.58 μg, respectively. In the 12th hour, both systems almost reached equilibrium, and the cumulative release amounts of OT from the nanoparticles in PBS and 10% rat serum were determined to be 7.34 and 19.35 μg, respectively. Similar release behaviors have been reported in previous studies.^20^

Although it has been known that IN-administered OT, which is a large molecule for the BBB, has beneficial effects on the brain, the desired amount of BBB passage rate, brain penetration, and half-life cannot be obtained for OT by this administration way.^18^ For this reason, researchers have chosen high doses of OT. But the side effects of the high doses of OT should also be considered. Therefore, the design of a suitable nanoparticle system for OT has recently been studied by Zaman et al.^20^ aiming at high effectiveness even at low doses. Poly(lactic-*co*-glycolic acid) (PLGA) and BSA were chosen as the base materials for the NPs, considering their advantages such as being biocompatible and biodegradable as well as being approved by the American Food and Drug Administration (FDA). And it was found that, of these, BSA is the most suitable base material in terms of release profile. In addition, the study aimed to provide a more efficient transition by binding these NPs to the receptors on the BBB with targeted ligands. As a result, they showed that the NP-coated OTs synthesized by conjugation to the transferrin ligand were one of the sufficient formulas to pass through the BBB with a size of 240 ± 0.72 nm. Oppong-Damoah et al.^19^ also showed the construction of in vitro OT-like large molecules for NP systems in the BBB model and again chose BSA as the base material in the NP system they constructed. Afterward, they applied NPs to mice and proved the efficiency of the NP transport system of OT in the brain by bioimaging methods. In a different study,^23^ the effects of nanoparticle-coated OTs were investigated by IN administration to mice with Dravet syndrome, which is a type of epilepsy syndrome induced by *SCN1A* gene mutation. They reported that this treatment has better effects on seizures and related social alterations compared to treatment with only OT by acting on OT receptors, which also supports the findings of Erfanparast et al.^26^

According to these data, it was understood that the safest and most effective way of transporting OT to the brain was to use BSA as the main base material. In addition, according to the high expression of the transferrin receptor in the nasal epithelium, the transferrin-bound NPs delivered intranasally can reach the BBB more effectively, as well as considering the transferrin receptors on the BBB.^19^ We aimed to obtain maximum efficiency by conjugating the transferrin ligand to the NP-OT system. The size of the prepared NP-OTs was 226 ± 14.4 nm, which is also consistent with previous studies. Therefore, our findings show that prepared NP-OTs are at an appropriate size for the effective passage through the BBB. Considering our OT release profile, dose adjustment of NP-OTs was applied by calculating less than 20 μg (19.35 μg) release in 24 h.

### Chronic Administration of Oxytocin and Oxytocin-Loaded Nanoparticles Protects Pentylenetetrazole-Induced Seizures

**Figure 3:**
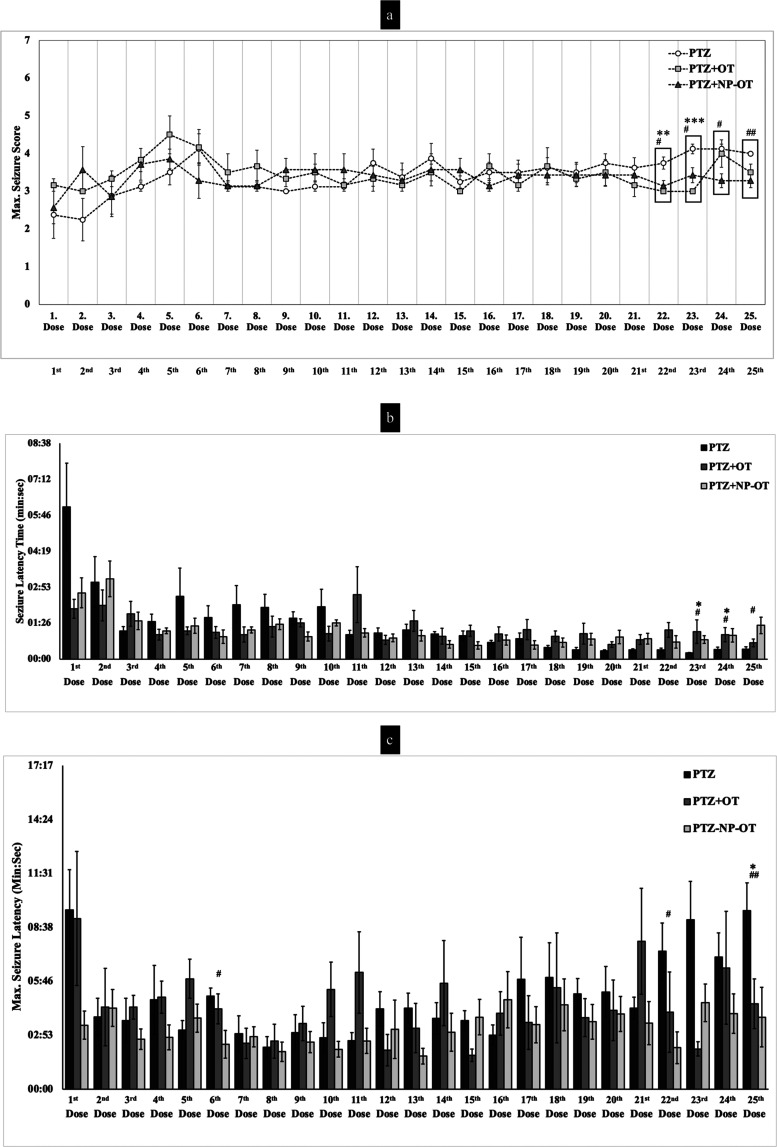
Calculated max seizure score (a), seizure latency (b), and max seizure latency (b) for each dose of PTZ. Statistical analyses were performed using the Kruskal–Wallis *H* test, followed by Dunn’s comparisons of selected group pairs: ^#^*P* and *P < 0.05, ^##^*P* and **P < 0.01, ***P < 0.001 compared to the PTZ group. Our supplementary dataset published by Sahin et al.^69^ also reported the detailed table for the max seizure score, seizure latency, and max seizure latency.

The animals that were injected with PTZ demonstrated a seizure activity during the 20 min observation period, whereas Ctrl groups showed no abnormal behavior after saline injections. All of the animals belonging to the PTZ group showed three subsequent convulsive seizures (>3 score) at the 25th dose, which indicates that those animals developed kindling induced by PTZ. Surprisingly, none of the animals, neither from PTZ + OT nor from PTZ + NP-OT groups, developed kindling at the 25th dose of PTZ injection (Figure 3a). These findings showed that both chronic IN OT and NP-OT administrations applied at low doses can provide resistance to the formation of the fully kindling model.

PTZ + OT and PTZ + NP-OT groups generally showed lower max seizure scores starting from the 12th dose, compared to the PTZ group. However, there was no statistical significance for the first 21 doses (Figure 3a). In contrast, a Kruskal–Wallis *H* test showed that there was a significant difference in the max seizure scores between the different treatments for the 22nd (χ^2^(2) = 9.898, *P* = 0.007), 23rd (χ^2^(2) = 13.458, *P* = 0.001), 24th (χ^2^(2) = 6.796, *P* = 0.033), and 25th (χ^2^(2) = 8.173, *P* = 0.017) doses. PTZ + OT and PTZ + NP-OT groups showed significantly lower max seizure scores at 22nd and 23rd doses, compared to the PTZ group according to Dunn’s post-hoc test (22nd dose: *P* = 0.004 and 0.015; 23rd dose: *P* < 0.001 and P 0.024, respectively, for the groups; Figure 3a). In addition, the PTZ + NP-OT group maintained the significance of lower max seizure until the last injection of PTZ (24th dose: *P* = 0.012 and 25th dose: *P* = 0.005; Figure 3a).

As for the seizure latencies, the PTZ group demonstrated a tendency for lower seizure latency during the PTZ injection procedure. Although OT administration resulted in a similar trend until the 7th dose, there was neither an increasing nor a decreasing trend for the following doses in terms of the seizure latency. A Kruskal–Wallis *H* test showed that there was a significant difference in seizure latencies between the different treatments for the 23rd (χ^2^(2) = 7.901, *P* = 0.019), 24th (χ^2^(2) = 7.617, *P* = 0.022), and 25th (χ^2^(2) = 6.189, *P* = 0.045) doses. A post-hoc Dunn’s test revealed that NP-OT administration increased the time of seizure latency for the 23rd, 24th, and 25th doses, and this was statistically significant compared to the PTZ group. (*P* = 0.01, 0.014, and 0.013, respectively). Further, OT administration increased this time significantly only for the 23rd and 24th doses compared to the PTZ group (*P* = 0.0305 and 0.0266; Figure 3b). Interestingly, a Kruskal–Wallis *H* test also showed that there was a significant difference in max seizure latencies for the 6th (χ^2^(2) = 6.154, *P* = 0.046), 22nd (χ^2^(2) = 6.553, *P* = 0.038), and 25th (χ^2^(2) = 8.106, *P* = 0.017) doses. A post hoc Dunn’s test showed that there was a decrease in the max seizure latencies on the 6th and 22nd doses for only the PTZ + NP-OT group (*P* = 0.015 and 0.01, respectively) and on the 25th dose for both PTZ + OT and PTZ + NP-OT groups (*P* = 0.031 and 0.009; Figure 3c). We did not observe any significant difference between PTZ + OT and PTZ + NP-OT groups in terms of max seizure scores and seizure latencies for any of the PTZ doses (Figure 3a–c).

The effects of exogenous administration of neurohypophysial hormones such as OT, arginine vasopressin, isotocin, and arginine vasotocin were observed in PTZ-induced seizures of zebrafish by Braida et al.,^27^ and it was shown that these hormones have anticonvulsant and neuroprotective effects in PTZ-induced seizures on zebrafish. Sala et al.^28^ studied the effects of seizures induced by PTZ on both mice with autism-like behavior (caused by the lack of OT receptor gene expression) and normal mice as a control group (not genetically manipulated). As a result, it was observed that the fluctuations that are seen during the myoclonic seizures increased in the control group, while fluctuations that are seen during the generalized tonic-clonic seizures increased in mice lacking OT receptors according to electroencephalogram (EEG) recordings. In addition, shorter seizure latency periods were observed in mice lacking the OT receptor. Furthermore, they emphasized that 250 μg/kg subcutaneous OT administration performed before subconvulsive PTZ injection reduced the spike fluctuations observed in the EEG and increased the latency periods in both mouse strains. However, statistically significant changes were seen in the mice lacking OT receptors but not in the control group. It was concluded that the reason the significant changes could not be obtained in the control group was due to the OT dose or the method of administration.^30^ Therefore, Loyens et al.^30^ aimed to study the dose–response effect of OT with two different doses (0.25 and 0.5 mg/kg) in PTZ-induced seizures in mice and showed that high-dose OT significantly reduced seizures. In another study, it was observed that intraperitoneally low-dose OT administration (40 nmol/kg) once a day for 5 days did not have a significant effect on seizures in rats, while high-dose OT administrations (80 and 160 nmol) significantly reduced seizures.^31^ Erfanparast et al.^26^ administrated OT directly to the CA1 region of the right and left hippocampus via intracerebroventricular way. These researchers have seen a significant change in seizures depending on the dose of OT, and they have also shown that OT and GABA_A_-benzodiazepine receptors are related to this antiepileptic effect.

In our study, a 20 μg dose of OT was chronically administered intranasally before PTZ injections to evaluate the effectiveness of OT by applying it at a relatively low dose compared to other studies. Before PTZ injections, 20 μg of chronic IN OT administration decreased max seizure scores at the 22nd and 23rd doses, while it increased the seizure latencies at the 23rd and 24th doses. Although it has shown that chronic OT administration at low doses can have positive effects on seizures in long term, its effects were not lasting according to our max seizure score and seizure latency results. We also showed that NP-OTs, which give less than 19.35 μg of OT release in 24 h in vitro, have a higher reduction in the seizure severity for the PTZ + NP-OT group when compared to the PTZ + OT group. In the PTZ + NP-OT group, max seizure scores were decreased at the 22nd, 23rd, 24th, and 25th doses, and seizure latencies were increased at 23rd, 24th, and 25th doses. To our knowledge, this is the first study to show that low-dose OT may have a positive effect on seizures as well as delay the development of kindling.

One of the limitations of our study is solely using male rats. There is evidence for sexually differentiated effects of vasopressin and OT in both humans and nonhuman animals when administered exogenously.^32^ It is also known that these neuropeptide systems are involved in the sex-specific regulation of social behavior in rodents and humans.^33^ Moreover, sexual dimorphism is described in human epilepsy.^34−36^ Also, some studies indicate that seizures and epilepsy can differ by gender in model organisms like rodents including the PTZ-kindling model.^37−39^ So the potential influence of sex as a biological variable remains to be addressed for the effects of OT/NP-OT administration on PTZ-kindling rats.

### Oxytocin-Loaded Nanoparticles Prevent Pentylenetetrazole-Induced Memory Alteration

The spatial working memory evaluation test (SWMET) is based on the urge to explore new places that naturally exist in rodents. With this feature, rodents exhibiting spontaneous displacement behavior in the Y-maze, which has three different arms at the same angle, are associated with spatial working memory. It is known that this event is neurobiologically related to different parts of the brain such as the hippocampus and the prefrontal cortex. For this reason, spatial working memory evaluation is widely used to investigate rodents’ memory.^24,40^ The PTZ-kindling model is known to have detrimental effects on cognitive functions such as spatial working memory.^41^

**Figure 4:**
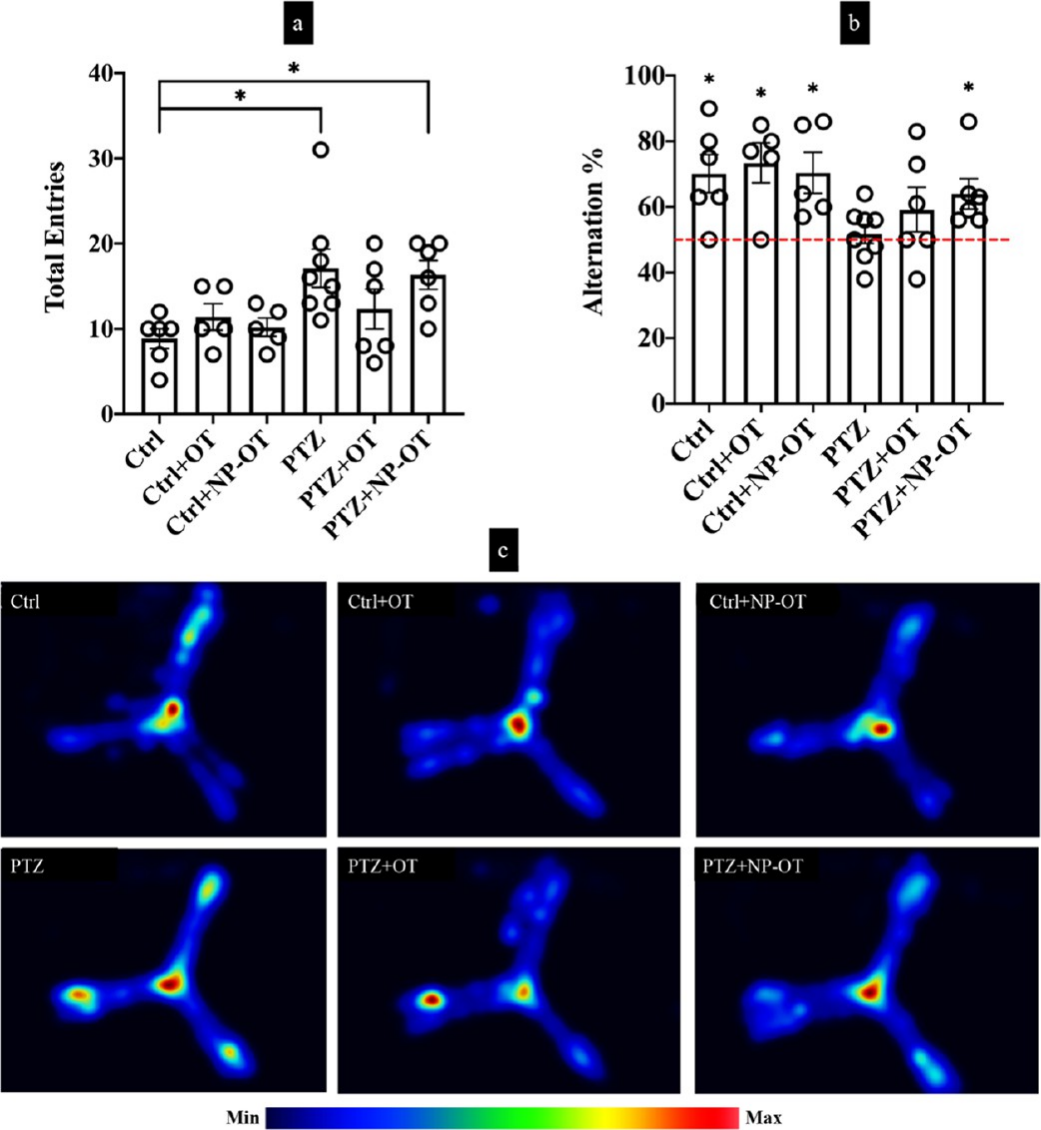
Behavior analysis by Y-maze. (a) PTZ (*n* = 7) and PTZ + NP-OT (*n* = 5) groups showed higher total entries to the Y-maze arms (*P < 0.05); however, the PTZ + OT (*n* = 5) group did not show a difference compared to Ctrl groups. (b) Only Ctrl (*n* = 5), Ctrl + OT (*n* = 4), Ctrl + NP-OT (*n* = 4), and PTZ + NP-OT groups had a higher percentage of spontaneous alternation above the 50% chance (*P < 0.05). PTZ and PTZ + OT groups did not reach the statistical significance for the 50% chance level. (c) Similarly, Ctrl groups and the PTZ + NP-OT group showed a similar average behavior pattern in Y-maze according to heatmaps; however, PTZ and PTZ + OT groups demonstrated an alteration of behavior. Statistical analyses were performed using one-way ANOVA, followed by Tukey comparisons of selected group pairs for total entries and a single sample *t*-test comparison to the chance level (50%) for alternation %.

The next day of the last injection of PTZ, all animals were evaluated for the SWMET in Y-maze. Ctrl, Ctrl + OT, and Ctrl + NP-OT showed spontaneous alternation % of 70 ± 5.88, 73.31 ± 6.04, and 70.27 ± 6.25, respectively, which all were above the 50% chance level and reached the statistically significance according to a single sample *t*-test (*t*(50) = 3.401, *P* = 0.019; *t*(50) = 3.853 *P* = 0.018; and *t*(50) = 3.244, *P* = 0.032, respectively), while this was not the case for spontaneous alternation % of PTZ and PTZ + OT groups (51.85 ± 2.79, *t*(50) = 0.662, *P* = 0.529 and 59.37 ± 6.79, *t*(50) = 1.380, *P* = 0.226, respectively; the results are presented as mean (*M*) ± standard error of mean (SEM); Figure 4b). However, the PTZ + NP-OT group showed no alteration in terms of spontaneous alternation %, which was 63.63 ± 14.31%, similar to the Ctrl groups (*t*(50) = 2.945, *P* = 0.032; Figure 4b). There was a significant difference between groups for the total number of arm entries as determined by one-way analysis of variance (ANOVA) (*F*(5,30) = 3.337, *P* = 0.015; Figure 4a). A Tukey post-hoc test revealed that the total number of arm entries for the PTZ group was significantly higher compared the Ctrl group (*P* = 0.026; Figure 4a). However, there was no significant difference between the other groups (Figure 4a).

When overall activities of the groups in the maze were investigated by heatmaps, there was a behavior change for the PTZ group compared to Ctrl groups. While all Ctrl groups tend to be active mostly in the middle zone of the Y-maze over the 8 min period, PTZ-kindled animals followed a different distribution of activity patterns. This pattern was seen as distributed on all distal arms of the Y-maze beside the middle zone. However, this behavior was changed for the PTZ + OT group to which the one distal arm was more preferred activity area as well as lower interest in the middle zone. Interestingly, the PTZ + NP-OT group exhibited this activity pattern on the Y-maze just as the Ctrl groups (Figure 4c).

In the present study, while 25 doses of PTZ injection decreased spatial working memory in PTZ and PTZ + OT groups, spontaneous alternation % for the PTZ + NP-OT group was not altered compared to the Ctrl groups. This indicates that chronic administration of NP-OT can have protective effects from PTZ-induced memory problems. Our heatmap findings in Y-maze also support the beneficial effects of NP-OT for the PTZ + NP-OT group.

### Oxytocin-Loaded Nanoparticles Have Neuroprotective Effects against Pentylenetetrazole-Induced Hippocampal Damage

**Figure 5:**
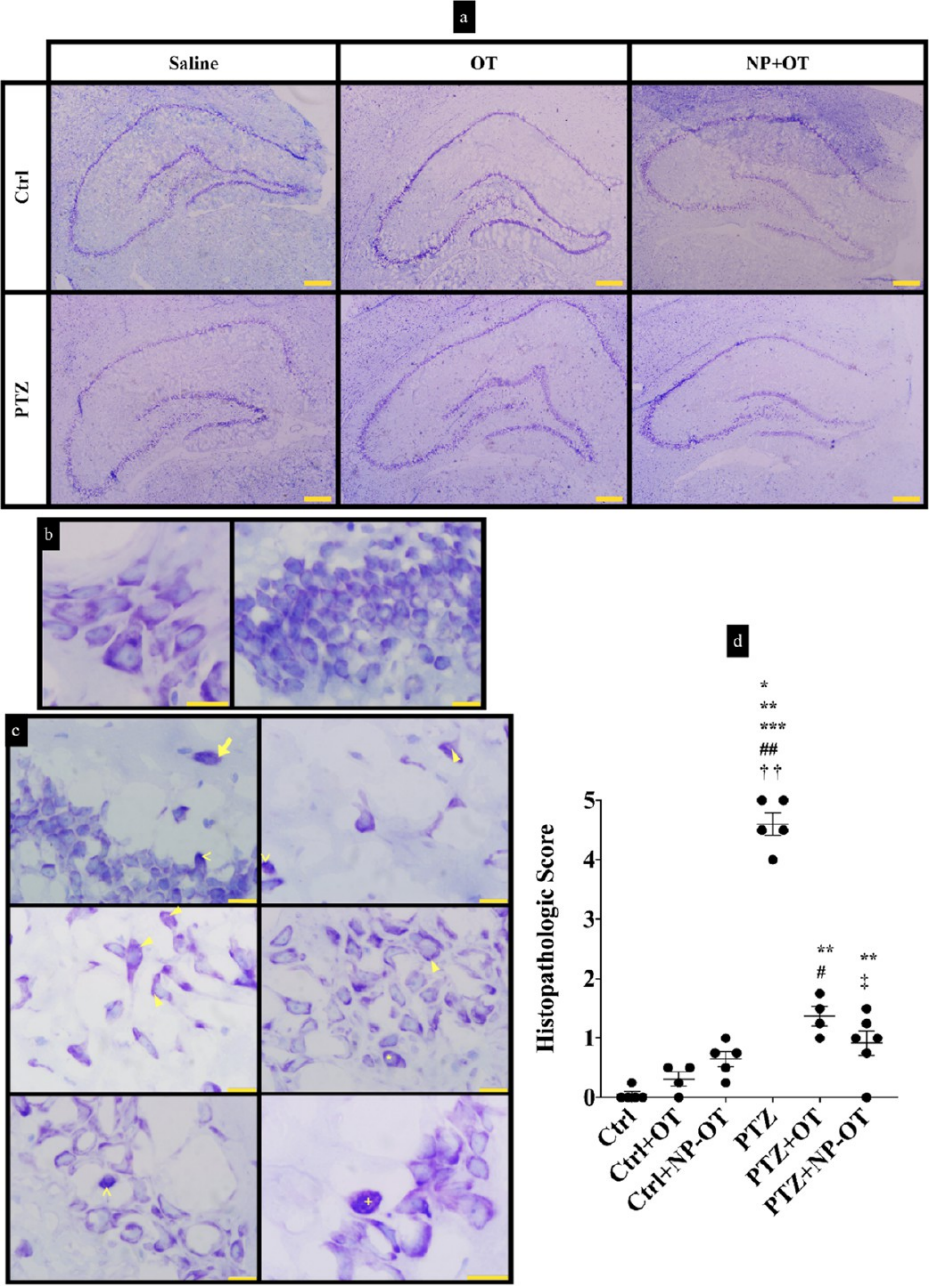
Histopathologic scoring on cresyl violet-stained hippocampus. (a) Representative microphotographs for the hippocampus of Ctrl and PTZ groups at low magnifications (4×, scale bars represent 100 μm). (b) Representative microphotographs for both pyramidal (left) and granular (right) neurons taken from the Ctrl group (*n* = 5) hippocampus (100×, scale bars represent 10 μm). (c) Most observed abnormal histomorphological findings in neurons during histopathologic scoring of the PTZ group (*n* = 5) hippocampus sections are cell fragmentation (arrow), cytoplasmic vacuolization (arrowhead), nuclear fragmentation (star), densely stained cell (<), and condensed cytoplasm (+) (100×, scale bars represent 10 μm). (d) Histopathologic scores from 0 to 5 are given for the hippocampus sections and these scores are compared between groups. PTZ group showed the highest pathological findings (*P < 0.05, **P < 0.01, and ***P < 0.001 compared to Ctrl, Ctrl + OT (*n* = 4), and Ctrl + NP-OT (*n* = 5) groups, respectively). However, the score of the PTZ + OT group (*n* = 4) was only higher than that of Ctrl and Ctrl + OT groups (**P < 0.01 and ^#^*P* < 0.05, respectively), whereas the score of the PTZ + NP-OT (*n* = 6) group was higher than that of the Ctrl group and lower than that of the PTZ group (**P < 0.01 and ^‡^*P* < 0.05, respectively). Statistical analyses were performed using the Kruskal–Wallis *H* test, followed by Dunn’s comparisons of selected group pairs.

When the hippocampus sections of experimental groups were compared in terms of histopathology, several neuronal damage indications were seen such as cell and nuclear fragmentations, cytoplasmic vacuolization, densely stained cells, and condensed cytoplasmic areas on CA and DG layers (Figure 5a–c). A Kruskal–Wallis *H* test also showed that there was a significant difference between the groups (χ^2^(5) = 22.889, *P* < 0.001). Then, a post-hoc Dunn’s test was applied to see comparisons of the selected group pairs. According to results, the PTZ group, which includes fully kindled animals, showed the highest histopathological evaluation with a score of 4.6 ± 0.18 and reached statistical significance compared to the Ctrl group (0.05 ± 0.05), the Ctrl + OT group (0.31 ± 0.11), and the Ctrl + NP-OT group (0.65 ± 0.16) (*P* < 0.001, *P* = 0.003, and *P* = 0.024, respectively; Figure 5d). However, OT administration parallel to PTZ injections resulted in a 1.37 ± 0.16 score, which is significantly higher than both Ctrl and Ctrl + OT groups (*P* = 0.003 and 0.032, respectively; Figure 5d); however, there was no significance compared to the PTZ group. On the other hand, the PTZ + NP-OT group demonstrated the lowest damage with a score of 0.92 ± 0.21 among PTZ-injected groups. The PTZ + NP-OT score was only higher than that of the Ctrl group but lower than that of the PTZ group, and this was statistically significant (*P* = 0.0083 and 0.0303, respectively; Figure 5d).

It is known that PTZ application causes hippocampal atrophy in rats in addition to selective neuronal loss and astrocytosis.^25^ Especially, chronic PTZ injection results in a higher number of both necrotic and apoptotic cells in the DG region. Therefore, perikaryal swelling and shrinking, chromatolysis, a decrease of Nissl bodies, and dark nucleus are seen in the hippocampal regions. Additionally, nuclear deformation, perikaryal outlines, and cisternae dilatations are shown by transmission electron microscopy (TEM) analyses in the PTZ-kindling model.^42,43^ Common findings such as disrupted myelin sheaths, degenerated axons, pre- and postsynaptic region abnormalities, and neuronal degenerations were seen by electron microscopy in different types of neurobiological pathologies.^44^

**Figure 6:**
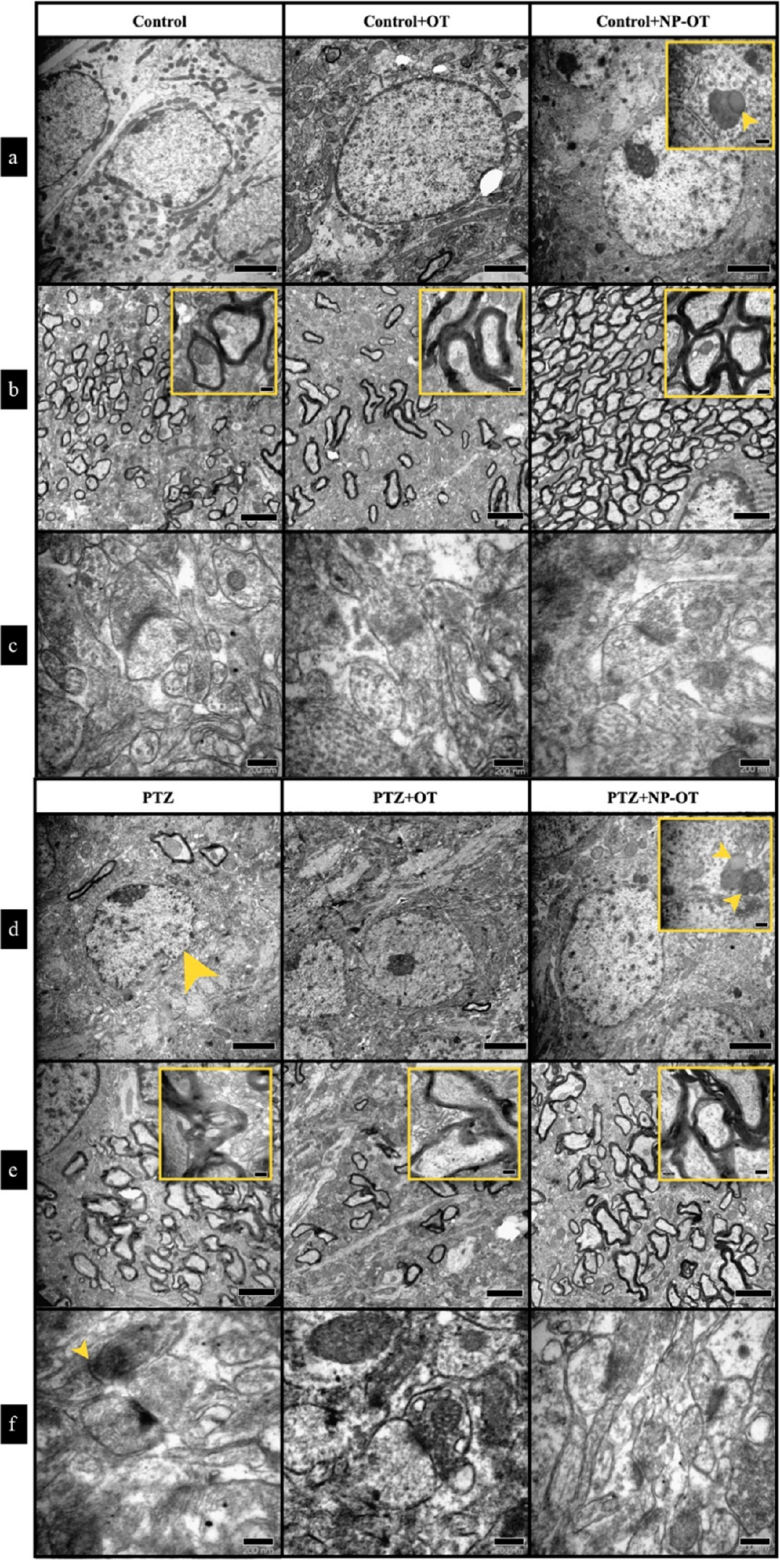
Transmission electron microscopic evaluation of the hippocampus. (a, d) Microphotographs of neurons seen in the anterior part of the hippocampus. NP-OTs were seen in both Ctrl + NP-OT and PTZ + NP-OT groups, which were mostly fused with lysosomes (arrowheads in insets). The PTZ group showed the most damaged structures such as (d) neuronal damage (arrowhead), (e) disorganized myelinization, and (f) abnormal synaptic terminals (arrowhead), whereas PTZ + OT and PTZ + NP-OT groups did not show damaged structures as severe as the ones observed in the PTZ group. Ctrl groups (*n* = 2) and PTZ groups (*n* = 3). Scale bar: 2 μm ((a, b, d, e); 200 nm for insets) and 200 nm (c, f).

**Figure 7:**
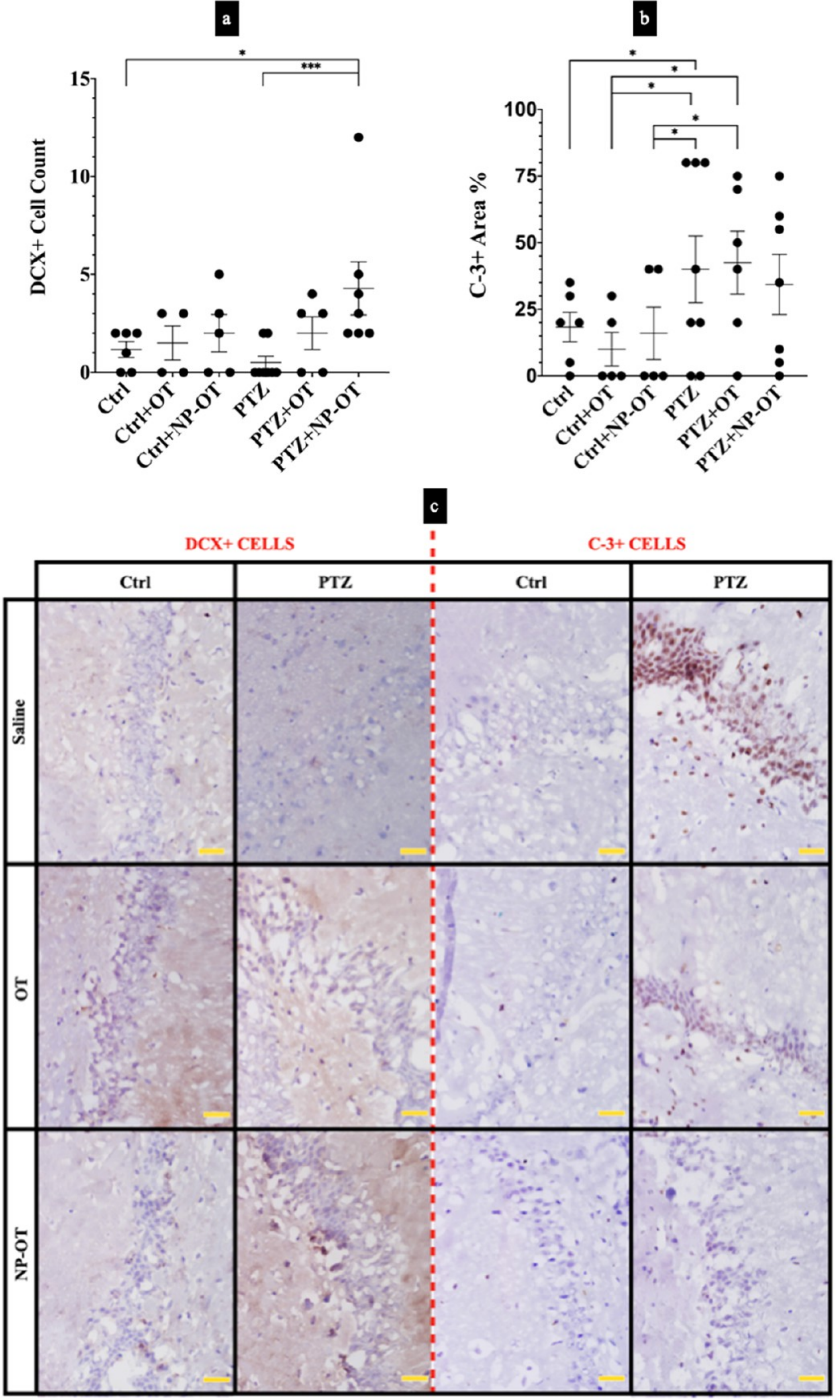
Comparison of neurogenesis and apoptosis in the hippocampus. (a) Neurogenesis (doublecortin (DCX) + cells) was higher in the PTZ + NP-OT group when compared to both Ctrl and PTZ groups (*P < 0.05 and ***P < 0.001). (b) Apoptosis (caspase-3 (C-3)++ cells), on the other hand, was increased in the PTZ group when compared to the Ctrl, Ctrl + OT, and Ctrl + NP-OT groups (*P < 0.05). However, the PTZ + OT group showed an increase in apoptosis when compared to Ctrl + OT and Ctrl + NP-OT groups only (*P < 0.05). The PTZ + NP-OT group showed no significant change in apoptosis levels. (c) Representative microphotographs of both DCX+ and C-3+ cells in hippocampus sections counterstained with Mayer’s hematoxylin. Scale bars represent 20 μm. Statistical analyses were performed using the Kruskal–Wallis *H* test, followed by Dunn’s comparisons of selected group pairs (Ctrl, *n* = 6; Ctrl + OT, *n* = 4; Ctrl + NP-OT, *n* = 4 and 5 for DCX+ and C-3+, respectively; PTZ, *n* = 8 and 6 for DCX+ and C-3+, respectively; PTZ + OT, *n* = 5; PTZ + NP-OT, *n* = 7 and 6 for DCX+ and C-3+, respectively).

The ultrastructure of the hippocampus supported histopathological results when observed with TEM. While Ctrl groups showed normal ultrastructural morphology of hippocampal neurons (Figure 6a–c), the PTZ group demonstrated the most severe damage such as nuclear fragmentation, disorganized myelinization, vacuolization areas, degenerated axons, and abnormal synaptic terminals, in addition to many apoptotic cells (Figure 7d–f). Although there were similar alterations in both PTZ + OT and PTZ + NP-OT groups (Figure 7d–f), the damage was not as severe as it was in the PTZ group. NP-OTs were also seen in both pyramidal and granular neurons of the hippocampus in both Ctrl + NP-OT and PTZ + NP-OT groups. The localization was only cytoplasmic and most observed NP-OTs were fused with lysosomes. The size and the shape of the NP-OTs in the cells were also consistent with our in vitro calculations (Figure 6a,d).

According to our histomorphologic results, cell fragmentation, cytoplasmic vacuolization, nuclear fragmentation, densely stained cells, and condensed cytoplasm were seen in PTZ groups in accordance with the previous data. Significantly less histopathological damage was seen in the PTZ + NP-OT group compared to the PTZ group. This suggests that the IN administration of NP-OTs has neuroprotective effects on hippocampal neurons. Nuclear fragmentation, disorganized myelinization, vacuolization, degenerated axons, and abnormal synaptic terminals were most severe in the PTZ group according to TEM results that supported our light microscopic observations. On the other hand, less damage was observed in the PTZ + NP-OT group. Thus, our histopathologic observations showed that NP-OT has neuroprotective effects against PTZ-induced damage. Previous studies showed that endocytosed nanoparticles such as BSA tend to accumulate in lysosomes.^45−47^

### Chronic Administration of Oxytocin-Loaded Nanoparticles Enhances the Adult Neurogenesis on Pentylenetetrazole-Injected Rat Hippocampus

Doublecortin (DCX) + cells as a neurogenesis marker were investigated in the hippocampus. As a result, DCX+ cells were counted to be 1.16 ± 0.4, 1.5 ± 0.87, and 1.75 ± 0.18 for Ctrl, Ctrl + OT, and Ctrl + NP-OT groups, respectively. A Kruskal–Wallis *H* test revealed that there was a significant difference between the groups (χ^2^(5) = 12.077, *P* = 0.034). DCX+ cells were counted to be 0.37 ± 0.26 for the PTZ group, which showed a decrease when compared to all of the other groups; however, the difference was not significant (*P* = 0.259, compared to the Ctrl group; Figure 7a) according to post-hoc Dunn’s test. Interestingly, we also observed that the PTZ + NP-OT group, which has a DCX+ cell count of 4.00 ± 1.19 was significantly higher than Ctrl and PTZ groups (*P* = 0.045 and <0.001, respectively; Figure 7a).

It is known that neurogenesis occurs continuously throughout the adulthood period in the DG region. Adult hippocampal neurogenesis consists of several developmental stages such as proliferation, differentiation, migration, axonal/dendritic targeting, and synaptic integration. This new neuron formation can be disrupted by various pathophysiological conditions. Toward the end of the differentiation phase, these cells begin to synthesize the DCX protein, and DCX+ cells can be seen in different morphologies and localization within the DG. Therefore, DCX is widely used in the literature for the detection of neurogenesis, and neurogenesis in the adult rat hippocampus can be screened by DCX labeling.^1,48,49^ It is suggested that the decrease in neurogenesis in human temporal lobe epilepsy (TLE) may lead to alterations in learning and memory. Chronic PTZ injections on rodents also reflect this phenomenon.^7^

In the present study, while the PTZ + NP-OT group showed a dramatic increase in neurogenesis, the Ctrl + NP-OT group did not exert an increase in neurogenesis. This suggests that NP-OTs have enhancing effects on neurogenesis, which is decreased due to the PTZ. The DCX protein is synthesized specifically at the migration and axonal/dendritic targeting stages of neurogenesis. Thus, these DCX+ cells cannot be considered as fully functional neurons that can be integrated into the hippocampus. Therefore, we encounter an important limitation of this study in terms of detecting neurons in different developmental stages of adult neurogenesis. We suggest that additional research should be performed to detect neurons in different stages, especially the mature neurons by labeling them with other appropriate postmitotic markers that are expressed in their final developmental stage of synaptic integration.^49^

### Chronic Administration of Oxytocin-Loaded Nanoparticles Prevents Pentylenetetrazole-Induced Hippocampal Apoptosis

Caspase-3 (C-3)+ cells as an apoptosis marker were also investigated in the hippocampus. A Kruskal–Wallis *H* test revealed that there was a significant difference in apoptosis rates between the groups (χ^2^(5) = 11.890, *P* = 0.036). A post-hoc Dunn’s test also revealed that C-3+ of the PTZ group was 40 ± 12.53%, which had the highest level compared to Ctrl, Ctrl + OT, and Ctrl + NP-OT groups (mean ± SEM = 18.33 ± 5.58%, *P* = 0.033; mean ± SEM = 10 ± 6.33%, *P* = 0.017, and mean ± SEM = 16 ± 9.8%, *P* = 0.026, respectively). While the PTZ + OT group showed a similar apoptosis rate, which was 42.5 ± 11.81%, NP-OT administration changed this rate to 34.29 ± 11.31% and did not reach significance compared to any of the groups including Ctrl groups (Figure 7b).

It is known that neuronal deaths are triggered by PTZ injections in many different regions of the brain, including the hippocampus via apoptosis using the C-3 pathway.^50,51^ It has previously been shown that OT protects neurons that undergo apoptosis via the C-3 pathway against various models in different types of neurons, including the hippocampal neurons.^52−54^

Our study shows that the apoptosis level of hippocampal neurons in the PTZ group was higher than that in the Ctrl groups. On the other hand, the PTZ + NP-OT group did not show a significant increase in apoptosis. This indicates that NP-OTs protect the hippocampal neurons from PTZ-induced apoptosis. It is also noteworthy that apoptosis and neurogenesis rates have a reverse correlation in neurobiological pathologies.^55^

### Pentylenetetrazole Injections Did Not Alter the Oxidative Stress Index (OSI) of Rat Serum

We also analyzed the serum of the animals in terms of oxidative stress. According to our results, the PTZ + NP-OT group showed the lowest level of total antioxidant status (TAS) which was 0.85 ± 0.12 mmol/L, while the other groups had similar results (0.85 ± 0.12, 1.02 ± 0.16, 1.03 ± 0.17, 1.16 ± 0.11, 1.03 ± 0.19 mmol/L for Ctrl, Ctrl + OT, Ctrl + NP-OT, PTZ, and PTZ + OT groups, respectively). On the other hand, the Ctrl group had the lowest level of total oxidant status (TOS), which was 32.72 ± 13.76 μmol/L, followed by the level of 35.79 ± 11.51 μmol/L for the PTZ + NP-OT group. The TOS values of Ctrl + OT, Ctrl + NP-OT, PTZ, and PTZ + OT groups were 53 ± 23.88, 50.17 ± 21.7, and 42.83 ± 4.49 μmol/L, respectively. Finally, the oxidative stress index (TAS/TOS/100, OSI) was compared between groups, and the PTZ + NP-OT group showed the lowest OSI. However, there was no statistical significance between any groups for neither TAS and TOS levels nor the OSI.

Different methods can be used to evaluate increasing levels of oxidative stress in epilepsy. For this purpose, glutathione and malondialdehyde are often used as markers. Significant changes with increased oxidative stress of these markers in both the hippocampus and serum were observed in rodents after PTZ injection.^56−59^ Another analysis used in the measurement of oxidative stress is TAS, which measures the total antioxidant capacity, and TOS, which measures the total oxidant capacity.^60,61^ While no change was observed in the TAS levels, the TOS levels were increased in the hippocampus after PTZ injection, and the OSI was increased significantly, according to the literature.^62^ However, there is no information about the change in TAS, TOS, and OSI values in the serum of rats after PTZ injection.

Our findings demonstrated that there was no significant difference between the TAS/TOS levels and the OSI in the serum of the Ctrl and PTZ groups. OT may have antioxidant effects in different organs;^63−66^ however, we can conclude that OT may not act as an antioxidant in the serum of PTZ-kindling rats. We only calculated TAS and TOS levels and the OSI after the 3rd day of the last PTZ injections. Thus, we suggest that those parameters should be calculated in serum during different periods of PTZ injections. We also believe that further research is needed to measure TAS and TOS levels and the OSI in other brain structures such as the frontal cortex and the hippocampus to understand the protective effects of OT.

## Conclusions

Recent research on the exogenous IN administration of OT has provided some beneficial effects on seizures. Current findings suggest that nanoparticle-carrying systems can enhance the OT therapeutic effects on the brain. However, there are very limited data on the effects of NP-OTs on epilepsy. Here, in this study, we reported that chronic administration of both OTs and NP-OTs protects pentylenetetrazole-induced seizures. Furthermore, we demonstrated in the current study that NP-OTs prevent memory alteration, hippocampal damage, and apoptosis while increasing adult neurogenesis.

All of these findings showed that more efficient results can be obtained using NP carrier systems applied through an IN way to increase the effectiveness of OT in the epileptic models. For this reason, further research is needed to elucidate the clinical potential of this method for the well-being of patients with epilepsy and related memory problems.

## Materials and Methods

### Synthesis of Oxytocin-Loaded Nanoparticles and Transferrin Conjugation

NP-OTs were synthesized from bovine serum albumin (BSA) and OT by a well-known procedure, which includes the desolvation method, followed by glutaraldehyde fixation (Merck, Germany, Cat# 820603).^19,20^ One milliliters of BSA (Sigma-Aldrich, MO, Cat#A2153) solution (30 mg/mL) and 0.5 mL of OT solution (2 mg/mL) were stirred for 30 min at 1200 rpm after the solution pH was set to 9. Subsequently, 2 mL (1 mL/min) of acetone was added dropwise until the solution became cloudy and stirred for 30 min. Afterward, 6 μL of 25% glutaraldehyde solution was added to the reaction solution, stirred for an hour, and centrifuged for 45 min at 14 000 rpm. The supernatant was discarded, while NP-OTs were collected and purified in distilled water. Finally, synthesized NP-OTs were lyophilized and stored at +4 °C for further processes.

Ligand conjugation to NP-OTs was performed using click chemistry agents with transferrin. Briefly, NP-OTs were first treated with 1 mL of 1-ethyl-3-(3-dimethylaminopropyl)carbodiimide (EDC) (Sigma-Aldrich, MO, Cat# E7750) and *N*-hydroxysulfosuccinimide (NHS) (Merck, Germany, Cat# 804518) solutions (1 mg/mL). Following this step, 1 mL of transferrin solution (1 mg/mL) was introduced and stirred for 4 h at room temperature. Then, the solution was centrifuged, and the purified pellet was separated. Finally, synthesized NP-OTs were lyophilized and stored at +4 °C for the IN treatment.

### Characterization and Particle Size Determination of Oxytocin-Loaded Nanoparticles

NP-OTs were characterized by Fourier transform infrared (FTIR) spectroscopy (Agilent, Cary 630), dynamic light scattering (DLS) (Malvern Instrument, Zetasizer Nano-ZS90, U.K.), and transmission electron microscopy (TEM) (JEOL, JEM-1011, Japan).

### OT Release Studies from Oxytocin-Loaded Nanoparticles

To investigate the OT release profile from NP-OTs, the release studies were carried out using a membrane permeability system. For this purpose, certain amounts NP-OTs were suspended either in PBS 7.4 or 10% (v:v) rat serum in PBS (pH 7.4) and then quickly placed inside the membrane (3500 molecular weight (MW) cutoff) and the outer part was filled with a certain amount of the same solution. The release medium was stirred at 100 rpm at 37 °C in an incubator, 2 mL of solutions was collected at certain intervals, and an equal volume of fresh solutions was added to the release solutions. The OT amount in the collected solutions diluted was determined by the enzyme-linked immunosorbent assay (ELISA).

Nanoparticle release samples in both PBS and 10% rat serum with PBS were measured to determine the OT levels using the OT ELISA kit (E-EL-0029, Elabscience Biotechnology Inc., TX). Briefly, 50 μL of samples or standards was placed in a 96-well plate coated with OT, and then 50 μL of biotinylated detection antibody was added to the plate. After 45 min of incubation (37 °C), the plate was washed three times and 100 μL of HRP conjugation was placed in the wells for a 30 min incubation period (37 °C). Next, the plates were washed and then filled with 90 μL of substrate solution and incubated (37 °C) for 15 min. Finally, a stop solution was filled in the plates, and the microplate was read under 450 nm using a microplate reader (AMR-100, Hangzhou Allsheng Instruments Co. Ltd., China). The final concentrations of the samples were calculated according to the manufacturer’s instructions using the four parameter logistic (4-PL) curve.

### Animal Experiments and Experimental Design

Male adult Sprague–Dawley rats (RRID: MGI:5651135) weighing 230.15 ± 4.43 g were obtained. The animals were kept in a room with a 12 h light–dark cycle with a temperature of 22–24 °C and relative humidity of 60–70%. The animals were fed ad libitum with standard food and provided free access to water. Ethical approval was provided for this study from Bezmialem Vakif University Animal Experiments Local Ethics Committee (BVU-HADYEK) with the given number of 2019/226 on 20th October 2020. All experiments were conducted during the light cycle in the Experimental Animals Laboratory of Bezmialem Vakif University (BEDEHAL), and the procedures were performed in accordance with the applicable national and international guidelines.

The animals were randomly divided into the following groups: control (Ctrl) (*n* = 6), Ctrl + OT (*n* = 5), Ctrl + NP-OT (*n* = 5), PTZ (*n* = 8), PTZ + OT (*n* = 6), and PTZ + NP-OT (*n* = 7).

### Development of Pentylenetetrazole Kindling

Grading subconvulsive PTZ (Sigma-Aldrich, MO, Cat# P6500) doses, which were 35 mg/kg from first injections, 37.5 mg/kg from second injections, and 40 mg/kg from third injections, were given to all PTZ groups. Therefore, PTZ dissolved in sterile 0.9% saline (NaCl) was injected into the animals of PTZ, PTZ + OT, and PTZ + NP-OT groups intraperitoneally for every 48 h. Similar to PTZ groups, all Ctrl groups were only injected with sterile saline.^67^

Video recordings were taken following 20 min of the PTZ injections for the observation of the animals’ behavior. Recordings of the observation period were used to score the seizures of the animals using Racine’s scale revised for PTZ-kindling rats.^68−70^ Seizure intensity stages were scored as sudden behavioral arrest and/or motionless staring (1), facial jerking with muzzle or muzzle and eye (2), neck jerks (3), clonic seizure in a sitting position (4), convulsions including clonic and/or tonic-clonic seizures while lying on the belly and/or pure tonic seizures (5), convulsions including clonic and/or tonic-clonic seizures while lying on the side and/or wild jumping (6), and death (7). Our supplementary dataset published by Sahin et al.^69^ also reported the video recordings for the seizure intensity stages. The maxi seizure score was assumed as the animal’s highest seizure intensity during the observation period. In addition, the starting point of the first seizure was assumed as the seizure latency, while the starting point of the first max scored seizure was assumed as max seizure latency and both were measured in min:sec. The animals that showed three subsequent convulsive seizures (>3) during the observation period were referred to as fully kindling.^25^

### Administration of Oxytocin and Oxytocin-Loaded Nanoparticles

The animals received treatment with either 30 μL of phosphate buffer saline (PBS), OT (20 μg) (RP10407, GenScript, NJ), or NP-OT (19.35 μg up to 24 h according to release profile, Figure 1) solutions 1 h before each PTZ injection using Hanson et al.’s^70^ protocol, with slight modifications. Thus, the animals’ heads were held up, and then PBS, OT, or NP-OT solutions were administered to the nostril using a micropipette.

### Spatial Working Memory Test

Spontaneous alternation behavior is considered to reflect the spatial working memory, which is a form of short-term memory. The animals were evaluated for the spatial working memory test (SWMT) in Y-maze, which has three identical arms labeled as A, B, and C with 120° angles.^71^ First, all animals were carried to the behavior room an hour earlier for their acclimatization. Second, the animals were placed separately in the distal part of the A labeled arm of the Y-maze, and video was recorded using the Noldus EthoVision XT (Noldus Information Technology, the Netherlands, RRID: SCR_000441). Spontaneous alternation behavior was defined as the entry into three arms on consecutive choices. An alternation number is defined as actual consecutive entries into all three arms, while the number of max potential alternation behaviors was calculated as the total number of arms entered minus 2. The spontaneous alternation percentage was calculated as the following formula *alternation number/max potential alternation × 100*.^72^ Additionally, heatmaps were obtained to investigate the animals’ pattern of behavior in the Y-maze during the experiment.

### Tissue Collection and Processing

Following the third day of SWMT, the animals were anesthetized, and blood samples were taken from the jugular vein. Next, blood samples were centrifuged at 2000 rpm for 10 min to separate serums, and all serums were kept at −80 °C for future analyses. The animals were sacrificed by the cardiac perfusion fixation method. First, PBS with Heparin solution (10 mM, pH 7.4, 200 mL) was injected into the anesthetized rats’ circulation, and subsequently, it was exchanged with a 4% paraformaldehyde fixation solution (pH 7.4, 300 mL) for whole-body fixation. Then, brain samples were taken. Left hemispheres were separated and used for the bright-field microscopic analysis. After incubation with sucrose solutions, coronal sections of the left hemispheres were obtained with a cryomicrotome from the mid-hippocampal plane. Right hippocampus tissues, which were isolated from the right hemispheres, were kept in a 4% glutaraldehyde solution for TEM analysis.

### Histological Observations

#### Cresyl Violet Staining

Cryosections from left hemispheres were washed with PBS and incubated in a 1% cresyl violet solution (pH 3.0, 37 °C) for 10 min. After washing with PBS, the sections were dehydrated and mounted. Histopathological investigations of the CA1 to CA4 regions and the DG layer of the hippocampus were scored by double-blinded histologists.^43,73−75^ Neurons were evaluated with a bright-field microscope (Olympus, BX61, Japan) under high magnification (1000×) to assess damage such as cell fragmentation, cytoplasmic vacuolization, nuclear fragmentation, densely stained cell, and condensed cytoplasm. Therefore, a semiquantitative histopathologic grading was used to ascertain relative neuron damage as follows: normal or no injury (0), rare neuronal injury (1), occasional neuronal injury (2), frequent neuronal injury (3), diffused neuronal injury (4), and severe neuronal injury (5).

#### Immunohistochemistry

A rabbit polyclonal antibody against doublecortin (DCX) (Biorbyt, U.K., Cat# orb457335, RRID: AB_2904159) was used to demonstrate neurogenesis on the subgranular zone of DG, and a rabbit polyclonal antibody against caspase-3 (C-3) (Sigma-Aldrich, MO, Cat# ab3623, RRID: AB_91556) was used to evaluate apoptosis (Table 1). The sections were washed with PBS, and H_2_O_2_ and blocking solutions were applied. Then, the sections were incubated with either primer anti-DCX (1:50) or C-3 antibodies (1:300) at +4 °C overnight. An HRP secondary antibody kit (HRP060, HRP-S-500, Zytomed, German) was used with 3,3′-diaminobenzidine (DAB) as the chromogen. Mayer’s hematoxylin was used as a counterstaining. During the protocol, the brain sections of the rat’s embryo and tonsil sections were used as either a negative or a positive control for primer DCX and C-3 immunohistochemistry, respectively. DCX+ neurons in the DG layer were counted. On the other hand, the average rate of C-3+ neurons in the hippocampus was calculated using Fiji software (RRID: SCR_002285).^76,77^ First, images were taken from CA1, CA2/3, and DG/CA4 regions under 40× objective from each hippocampus slide. Second, color deconvolution was applied to the images to split their color channels into blue (for hematoxylin), brown (for DAB), and white. Then, a threshold was applied on brown color channels to select only stained areas. Lastly, a measure was used to obtain the percentage of the stained area.

**Table 1:** Antibody List

antibody name	immunogen	manufacturer/RRID number/species/clonality	concentration
anti-doublecortin (DCX)	KLH-conjugated synthetic peptide derived from human DCDC2	orb457335/AB_2904159/rabbit/polyclonal	1:50
anticleaved caspase-3 (C-3)	cleavage-specific synthetic peptide sequence (proprietary); epitope: active (cleaved) form	ab3623/AB_91556/rabbit/polycolnal	1:300

#### Transmission Electron Microscopy

NP-OTs dissolved in 70° ethanol (1:1) were placed on formvar-coated copper grids to confirm the sizes and observe the morphologies of NP-OTs under the TEM.

After the fixation of the right hippocampus with a glutaraldehyde solution, tissues were postfixed with osmium tetroxide, dehydrated with grading ethanol series, incubated with propylene, and embedded in araldite blocks. Semithin sections were taken with an ultramicrotome (Reichert UM3) and the sections were stained with toluidine blue for localization of the neurons. Then, ultrathin sections were taken on copper grids with an ultramicrotome. Grids were stained with uranyl acetate and lead citrate. Then, grids were observed under a TEM (JEOL, JEM-1011, Japan) by double-blinded researchers.

### Oxidative Stress Assay

Total antioxidant status (TAS) and total oxidant status (TOS) (Rel Assay Diagnostic, Turkey) were calculated from serums according to the manufacturer’s instructions. The oxidative stress index (OSI) was calculated using the TAS/TOS/100 formula.

### Statistical Analyses

Data were analyzed using R.^78^ All data were tested for normality using the Kolmogorov–Smirnov test and then analyzed using appropriate statistical tests. One-way ANOVA, followed by the Tukey multiple comparisons test, was used when data showed no violated homogeneity. Otherwise, a nonparametric Kruskal–Wallis *H* test, followed by Dunn’s multiple comparisons test, was used for multiple comparisons. We followed Armstrong’s^79^ suggestion for a low sample size. A one-sample *t*-test was carried out in the Y-maze test to compare each condition with the chance level (50%) as previously reported.^80^ Group sizes were determined by power analyses with GPower 3.1.9.7.^81^*P* < 0.05 was accepted as statistically significant. The results are presented as the mean (*M*) ± standard error of the mean (SEM).

## Abbreviations

CA: cornu ammonis
DG: dentate gyrus
CNS: central nervous system
TLE: temporal lobe epilepsy
AED: antiepileptic drug
OT: oxytocin
BBB: blood–brain barrier
IN: intranasal
PTZ: pentylenetetrazole
NP-OT: oxytocin-loaded nanoparticle
DCX: doublecortin
C-3: caspase-3
DAB: 3,3′-diaminobenzidine
TEM: transmission electron microscopy
TAS: total antioxidant status
TOS: total oxidant status
OSI: oxidative stress index
